# A Much Underpaid Profession

**Published:** 1920-02-28

**Authors:** 


### THE EDITOR'S LETTER-BOX.

**Correspondence on all subjects is invited, but we cannot in any way be responsible for the opinions expressed by our correspondents, who must give their name and address as a guarantee of good faith, but not necessarily for publication. Correspondents are reminded that brevity of style and conciseness of statement greatly facilitate early insertion.]**

### A MUCH UNDERPAID PROFESSION.

*To the Editor of* The Hospital.

Sir,—Will you permit me a small space in your valuable and widely read paper?

At a meeting of the Chelsea Guardians, held a few weeks ago, it was decided to increase the salaries of all the nursing staff of the Chelsea Infirmary.

Now, Sir, everyone in these days is feeling the burden of high prices of the bare necessities of life, and our nurses are affected just the same as those who work in our factories and follow other callings. These gentlemen, after due consideration, decided to give an all-round advance of two pounds per year, or 9½d. per week! Surely some mistake.

Is not this a scandalous insult to the nursing profession, as this mere pittance is not given to a few probationers only, but to some who have put in anything from five to ten years.

There are many fully-trained nurses who receive the wickedly low pay of about £30 to £40 per year, plus a 20 to 30 per cent. bonus to meet the extra cost of living, which is still over 100 per cent. Do these gentlemen know what training and knowledge nurses have to acquire, also what this training costs in the examination fees? Where would our doctors be without the efficient care of these 1920 Florence Nightingales?

Sir! Cannot something be done to raise the pay of this ever-needful profession in accordance with their skill, which they can justly claim? Guardian.

